# Targeted next-generation sequencing for the detection of ciprofloxacin resistance markers using molecular inversion probes

**DOI:** 10.1038/srep25904

**Published:** 2016-05-13

**Authors:** Christopher P. Stefan, Jeffrey W. Koehler, Timothy D. Minogue

**Affiliations:** 1 United States Army Medical Research Institute of Infectious Disease, Diagnostic Systems Division, Fort Detrick, Maryland, 21702, United States of America

## Abstract

Antibiotic resistance (AR) is an epidemic of increasing magnitude requiring rapid identification and profiling for appropriate and timely therapeutic measures and containment strategies. In this context, ciprofloxacin is part of the first-line of countermeasures against numerous high consequence bacteria. Significant resistance can occur via single nucleotide polymorphisms (SNP) and deletions within ciprofloxacin targeted genes. Ideally, use of ciprofloxacin would be prefaced with AR determination to avoid overuse or misuse of the antibiotic. Here, we describe the development and evaluation of a panel of 44 single-stranded molecular inversion probes (MIPs) coupled to next-generation sequencing (NGS) for the detection of genetic variants known to confer ciprofloxacin resistance in *Bacillus anthracis, Yersinia pestis,* and *Francisella tularensis*. Sequencing results demonstrate MIPs capture and amplify targeted regions of interest at significant levels of coverage. Depending on the genetic variant, limits of detection (LOD) for high-throughput pooled sequencing ranged from approximately 300–1800 input genome copies. LODs increased 10-fold in the presence of contaminating human genome DNA. In addition, we show that MIPs can be used as an enrichment step with high resolution melt (HRM) real-time PCR which is a sensitive assay with a rapid time-to-answer. Overall, this technology is a multiplexable upfront enrichment applicable with multiple downstream molecular assays for the detection of targeted genetic regions.

More than 2 million people in the United States succumb to antibiotic resistant (AR) infections annually adding significant fiscal burden to the healthcare system due to prolonged treatments, extended hospital stays, and increased mortality rates^1^. The emergence of organisms such as carbapenem-resistant enterobacteriaceae, vancomycin-resistant enterococcus, and drug-resistant *Neisseria gonorrhoeae* highlight the need for more efficient measures to diagnose and treat AR organisms^1^. These measures would help to stop transmission as well as evolution of future multidrug-resistant strains through better antibiotic stewardship. Further, better diagnostics are even more critical when addressing biothreat agents where inadvertent creation of resistant organisms through overuse of antibiotics could have dire consequences.

Ciprofloxacin, a member of the quinolone family of antibiotic drugs, is a first-line therapy for numerous bacterial pathogens including high consequence biothreat agents^2^. Ciprofloxacin targets the bacterial type II topoisomerases, DNA gyrase, and topoisomerase IV^3^ by covalent linkage to conserved sites on these enzymes, termed quinolone-resistance determining regions (QRDR), resulting in permanent double stranded breaks and cell death. While highly effective against susceptible bacteria, high levels of drug resistance are readily acquired through genetic variants in the targeted genes^2^,^4^. In addition, fluoroquinolone resistance can also result from a decrease in drug accumulation through porin loss and overexpression of efflux pumps^3^,^5^. The high consequence pathogens *Bacillus anthracis, Yersinia pestis,* and *Francisella tularensis* are not naturally resistant to ciprofloxacin; however, serial passage of these organisms in the presence of ciprofloxacin quickly results in mutations in the QRDR, conferring high-level resistance^4^,^6^,^7^,^8^.

Diagnostic tools identifying QRDR genetic variants are essential for both timely and appropriate therapeutic measures as well as managing the spread of drug-resistant bacteria. Ideal diagnostics should be rapid, sensitive, inexpensive, and provide information such as strain-type, virulence prediction, and AR profile. Compared to current molecular diagnostics such as real-time PCR, next-generation sequencing (NGS) will potentially better fulfill these requirements while providing information beyond the identity of the etiologic agent^9^ including: species level identification, resistance profiling, genomic epidemiology, and microbial forensics^9^,^10^,^11^.

Currently unbiased metagenomic studies are expensive and bioinformatically challenging with poor clinical sensitivity^9^,^10^,^12^. Targeted sequence amplification strategies potentially mitigate these challenges^13^,^14^. Previous studies using techniques such as padlock and molecular inversion probes (MIP) as upfront enrichment steps for use with NGS showed the specificity and multiplexability of these techniques^13^,^14^,^15^,^16^,^17^. In this context, “gap-fill” molecular inversion probes, when combined with NGS, allow for the capture and evaluation of substantially more genetic information^18^,^19^,^20^,^21^. Specifically, after gap filling and probe circularization by a polymerase and ligase, the capture sequence can be amplified with the universal primer pair and prepared for NGS (Fig. 1). Recently, evaluation of the MIP platform for strain typing extended spectrum beta-lactamase producing *Escherichia coli* correlated well with the current diagnostic standard amplified fragment length polymorphism technique^21^. Similarly, studies showed MIPs are capable of detecting and distinguishing filovirus species for broad pathogen screening and biosurveillance^20^.

Here, we characterized the use of MIPs as an upfront enrichment step for the detection of genetic variants conferring ciprofloxacin resistance in high consequence pathogens *B. anthracis, Y. pestis,* and *F. tularensis* using NGS and high resolution melt (HRM) real-time PCR as downstream molecular detection technologies.

## Results

Mutations that result in ciprofloxacin resistance occur in the QRDR of genes that encode DNA gyrases and topoisomerase IV^3^,^4^. To test the viability of MIPs as a strategy for ciprofloxacin resistance detection, we designed complimentary probe arms (Supplementary Table S1) to flank known sequence variants causing resistance in the biothreat agents *B. anthracis* ΔANR, *Y. pestis* Kim5 and *F. tularensis* Schu4. Assay performance assessing the 44 pooled probes used whole genome amplified (WGA) DNA from wild-type strains to test across a broad range of input DNA concentrations.

**Figure 2:**
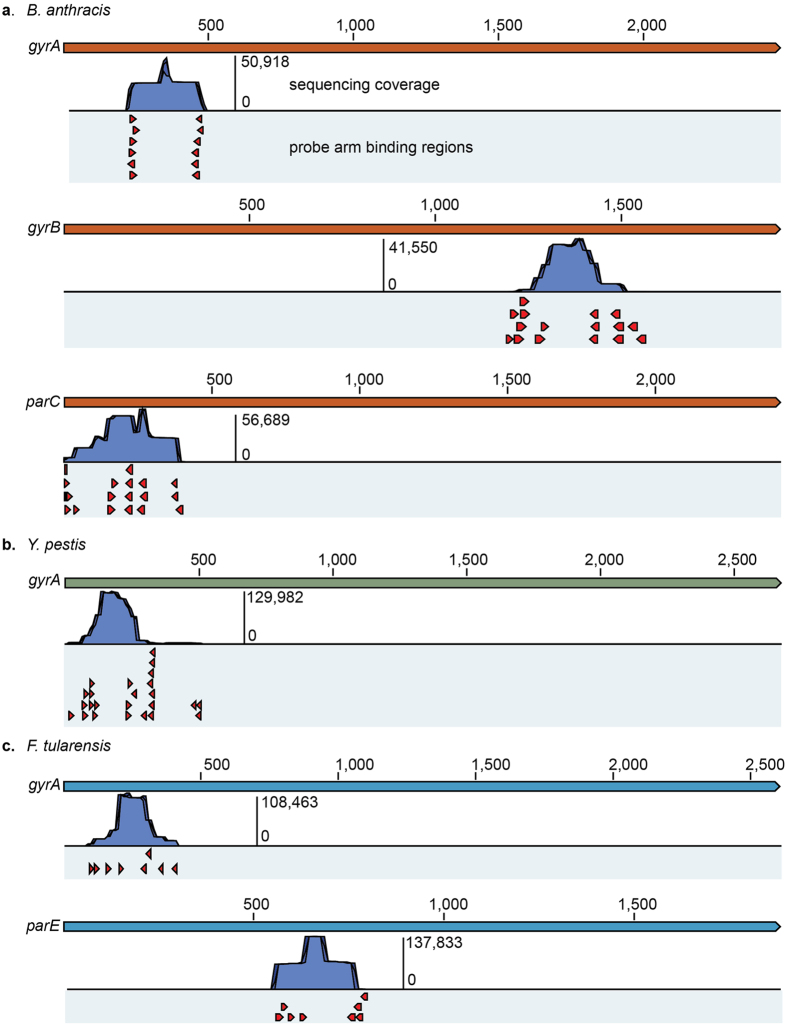
Sequencing reads generated from MIPs map to targeted capture regions. Pooled MIPs were tested against WGA DNA from wild-type (**a**) *B. anthracis*, (**b**) *Y. pestis,* and (**c**) *F. tularensis.* The depth of coverage of sequencing reads mapped against reference genes are shown in blue. Probe arms are represented by red arrows to demonstrate expected capture regions. Not all probe arms are represented.

**Figure 3:**
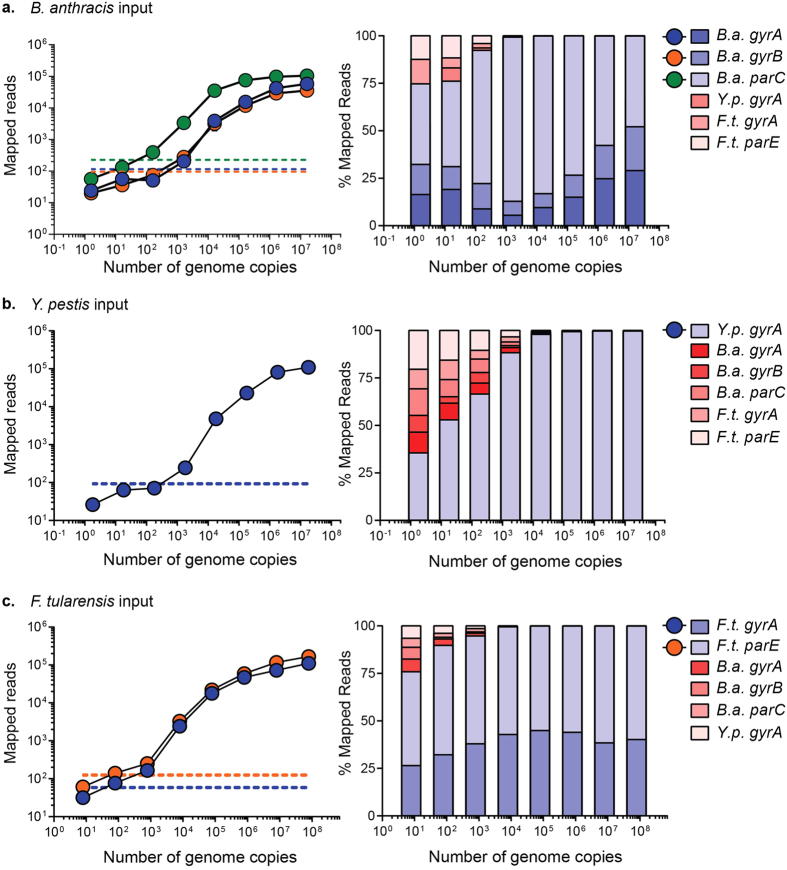
Preliminary sequencing results for MIPs on wild-type DNA. Pooled MIPs were tested against dilutions of WGA DNA from wild-type (**a**) *B. anthracis*, (**b**) *Y. pestis,* and (**c**) *F. tularensis.* All samples were dual indexed and pooled before sequencing on the Illumina MiSeq platform. Sequencing reads were mapped to reference genes and total mapped reads (left graph) plotted versus the total number of genome copies is represented (The scale of the X and Y axes are Log 10). Error bars represent the standard deviation of three replicates. Cutoffs are shown for each gene as dashed lines. The average percentage of mapped reads for each reference gene at each number of genome copies was plotted (right graph) as a stacked bar chart.

Superimposing sequence reads with the reference gene and probe arms demonstrated the depth of coverage for the target region of interest (Fig. 2). Sequencing reads which mapped to the six targeted QRDR reference genes plotted against total number of genome copies, calculated from DNA input concentrations, demonstrated a sigmoidal dose-response with a linear range beginning around 1000 genome copies for each gene mapped (Fig. 3). Often in pooled NGS runs dual-indexed sequencing reads will be misidentified during demultiplexing resulting in incorrect read mappings between samples known as sample bleeding^22^,^23^. To account for misidentified reads, non-template controls were used to define a background and are termed throughout the paper as the “cutoff”. The cutoff was determined as the average number of sequencing reads mapped to the reference gene in the non-template controls plus three times the standard deviation. Pearson coefficients demonstrated high degrees of correlation between target regions suggesting similar capture efficiencies (Supplementary Table S2). Total mapped reads composition, shown as a percentage of all mapped reads for each reference gene, indicated that decreases in total input DNA resulted in decreased correctly mapped reads (Fig. 3). Above the cutoff of the assay 80% or more of the sequencing reads mapped correctly. Most of the sequencing reads at or below the cutoff remained unmapped for the reference targets (Supplementary Figure S1). Indeed, at the cutoff, the low percentage (3%) of the total sequencing reads mapped to reference genes gradually increased to more than 90% of total reads at higher input concentrations (Supplementary Figure S1).

### Analytical limit of detection (LOD); performance and confirmation

**Figure 4:**
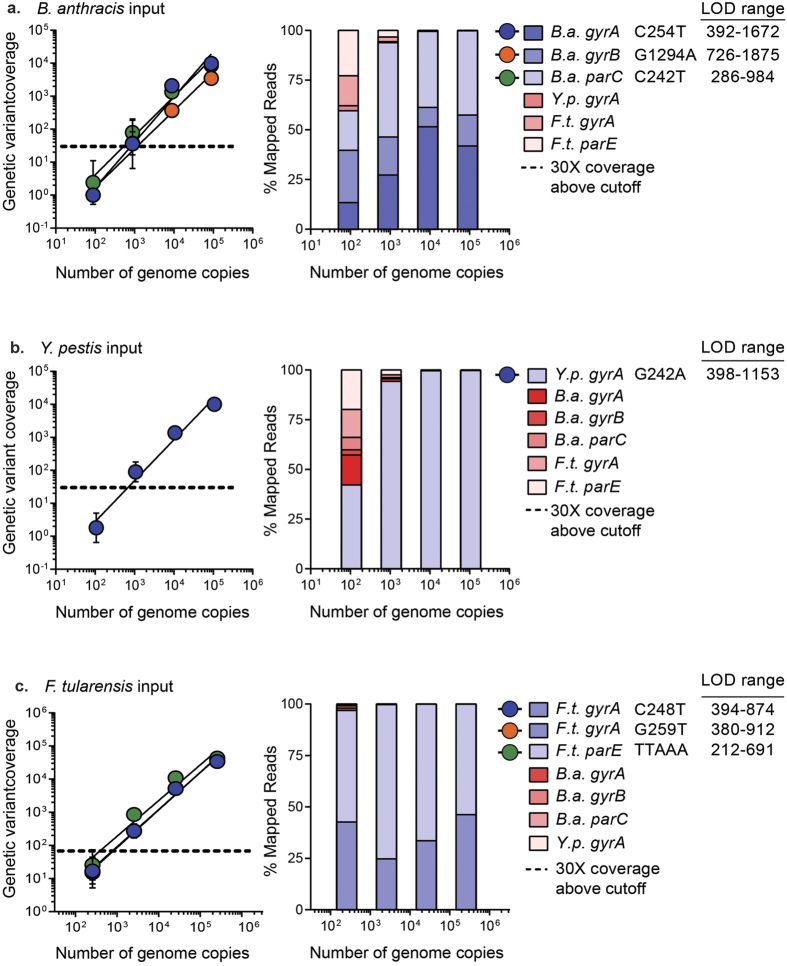
LOD for ciprofloxacin resistance conferring genetic variants. Pooled MIPs were tested against 10-fold dilutions of extracted DNA from ciprofloxacin resistant (**a**) *B. anthracis*, (**b**) *Y. pestis,* and (**c**) *F. tularensis.* All samples were dual indexed and pooled before sequencing on the Illumina MiSeq platform. Variance analysis was performed on mapped sequencing reads to calculate coverage at the indicated genetic variants. The genetic variant coverage was plotted against the total number of genome copies (The scale of the X and Y axes are Log 10). The LOD range was determined based off of the standard error of the linear regression line. Error bars represent the standard deviation of three replicates. The average percentage of mapped reads for each reference gene at each number of genome copies was plotted (right graph) as a stacked bar chart.

Capture of target genes demonstrated a preliminary cutoff for mapped reads above background levels in wild type cultures; however, the intended application of these probes was to detect ciprofloxacin resistance mutations. To test sequence coverage of the targeted genetic variants, we assessed strains with mutations known to confer resistance to ciprofloxacin^4^. Because WGA may incorporate bias^22^, these experiments used non-amplified whole genome extracted DNA to more accurately determine the assay LOD. Linear regression was utilized to fit data to a line and determine the point at which each genetic variant had 30X read coverage above the cutoff as determined by non-template controls^24^ (Fig. 4). This crossing point was determined to be the assay LOD. An LOD range was given for each genetic variant based on the standard error of the linear regression. In this context, each genetic variant fell within ranges of detection between 300 and 1200 input genome copies similar to the cutoff levels determined earlier. As demonstrated previously, the percentage of correctly assigned reads was between 80–100% at concentrations near or above the LOD (Fig. 4).

**Figure 5:**
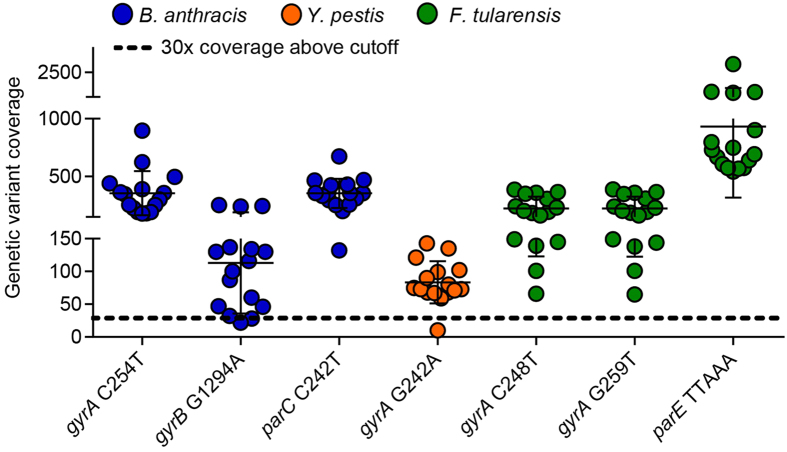
Confirmation of LOD for ciprofloxacin resistance conferring genetic variants. Pooled MIPs were tested against DNA from strains defined above at an input of 1.5× the upper limit of the calculated LOD range. All samples were dual indexed and pooled before sequencing on the Illumina MiSeq platform. Variance analysis was performed on mapped sequencing reads to calculate coverage at the indicated genetic variant with coverage of all 16 replicates represented along with the mean and SD.

Diagnostic assays require statistically reproducible LODs for clinical applicability. To confirm LOD performance, we used 1.5x the upper LOD range to demonstrate assay reproducibility. Specifically, these analyses tested detection of *B. anthracis, Y. pestis*, and *F. tularensis* genetic variants at 2813, 1729, and 1368 total input genome copies, respectively (Fig. 5), across 16 replicates. The number of mapped sequencing reads demonstrated high reproducibility between experiments with the total read coverage at each genetic variant falling near or above the 30× coverage line. No replicate tested showed zero coverage at a given location.

### Mock clinical performance

**Figure 6:**
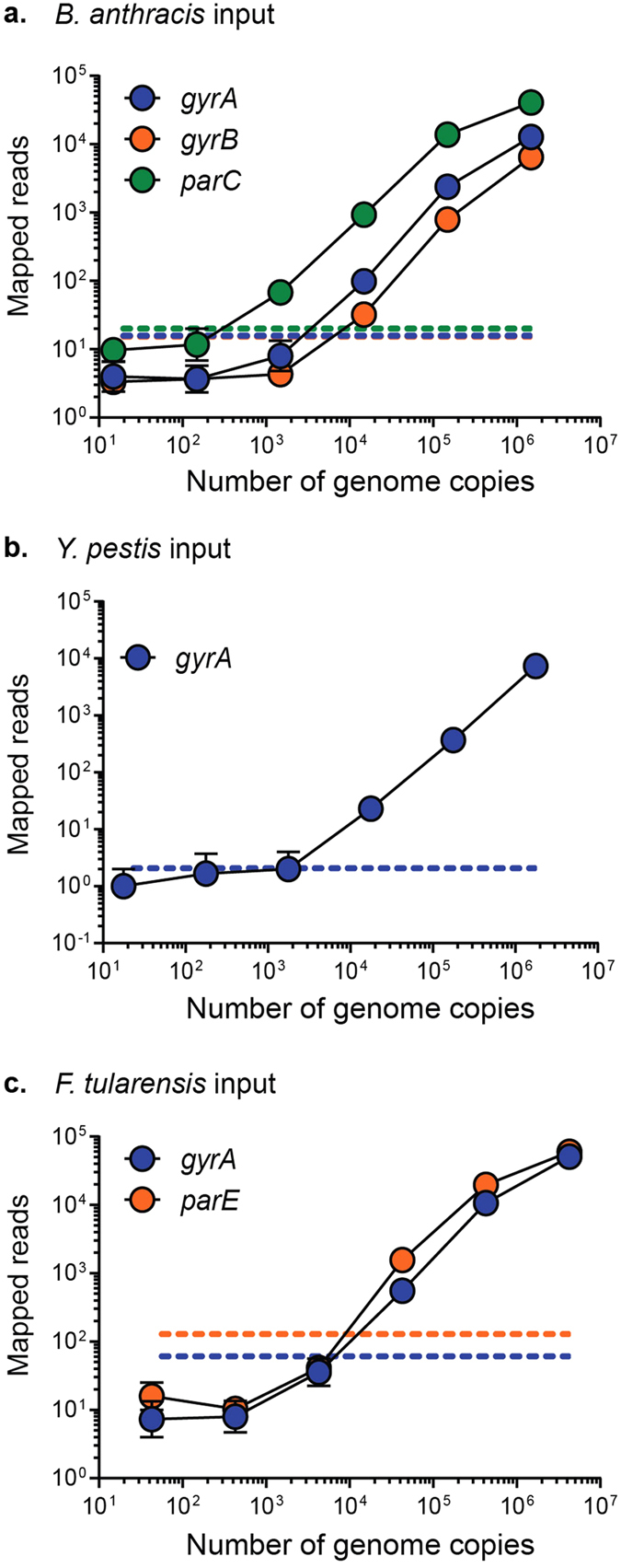
Performance of MIPs in a complex matrix. Extracted whole blood samples spiked with WGA DNA from ciprofloxacin resistant (**a**) *B. anthracis*, (**b**) *Y. pestis,* and (**c**) *F. tularensis* were tested with pooled MIPs. All samples were dual indexed and pooled before sequencing on the Illumina MiSeq platform. Sequencing reads were mapped to reference genes and total mapped reads plotted versus the total number of genome copies is represented (The scale of the X and Y axes are Log 10). Error bars represent the standard deviation of three replicates. Cutoffs are shown for each gene as dashed lines.

The ability to detect infectious agent directly from a patient sample is essential when evaluating new diagnostic tests. To determine assay performance over a broad range of input genomes in a complex sample matrix, we isolated DNA from whole blood spiked with WGA bacterial DNA. The MIP panel was tested on these samples to demonstrate assay performance in the presence of human genome. To directly compare the results to previous experiments the number of input genomes per reaction was calculated from ng of DNA per ml of blood assuming 100% extraction efficiency during sample processing (Fig. 6). As previously described, non-template controls were used to determine cutoff values for each gene. The number of input genome copies required for sequencing reads above background was approximately 10-fold higher than that of pure bacterial samples. Similar to the analytical analysis Person correlations demonstrated significant correlation between target regions suggesting similar capture efficiencies (Supplementary Table S2).

### High Resolution Melt analysis

**Figure 7:**
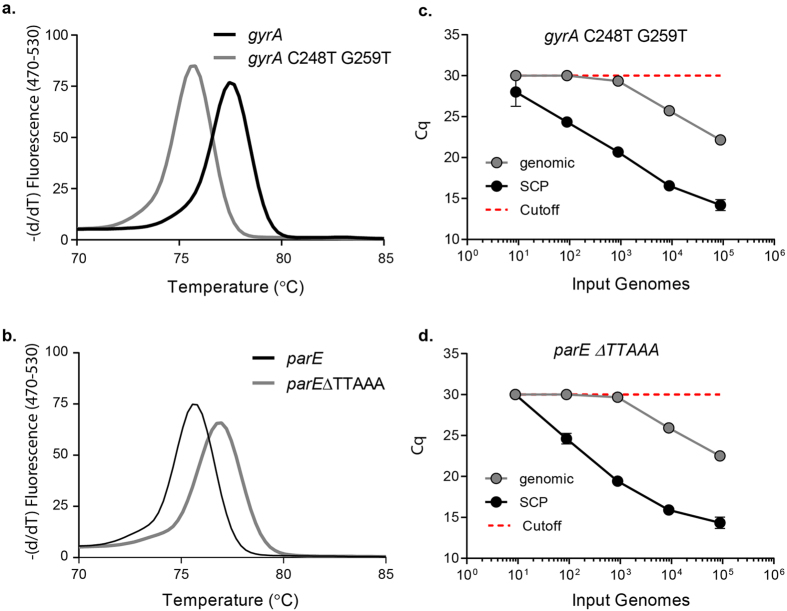
MIP amplicon detection with HRM real-time PCR. High resolution melt primers for *F. tularensis* (**a**) *gyrA* C248T G259T and (**b**) *parE* ΔTTAAA were tested on amplicons generated from samples in Fig. 3. Data curves are represented as HRM melting peaks. HRM Cq comparisons of genomic DNA and amplicons resulting from the MIP protocol at the same input genomic DNA for (**c**) *gyrA* C248T G259T and (**d**) *parE* ΔTTAAA are shown (The scale of the X axes are Log 10). Each curve represents the average of 3 replicates. Error bars represent the standard deviation of three replicates.

Sequencing data showed targeted amplification coupled to NGS was functional for determining ciprofloxacin resistance conferring genetic variants; however, this NGS strategy sacrifices cost, time-to-answer, and sensitivity for the benefit of a high degree of target multiplexability. In cases of limited sample volume and a large number of potential targets, real-time PCR or HRM real-time PCR screening is not feasible. A multiplexed upfront amplification strategy could be used to amplify targets of interest allowing for screening with these low cost, quick time-to-answer singleplex assays. To test MIPs applicability as a target amplification technique for other molecular assays, we assessed HRM assays targeting ciprofloxacin resistant genetic variants with amplicons generated by the MIP protocol. Detection of two *gyrA* mutations and a *parE* deletion in *F. tularensis*^4^ using the MIP protocol as an upfront target enrichment demonstrated differences between wild-type and ciprofloxacin resistant strains (Fig. 7a,b). Melting peaks with the *gyrA* C248T G259T mutation had an overall decrease in melting temperature, and the *parE* ΔTTAAA mutation had an increase in melting temp due to the increase in GC content. Furthermore, the use of MIPs significantly amplified target sequences based on Cq values for HRM experiments performed with purified genomic DNA versus amplified MIP DNA (Fig. 7c,d). Specifically, MIPs significantly reduced detection above background for the HRM analysis even after a 1:10,000 dilution of the MIP amplified material was required for the assays to fall within the optimal Cq range (Fig. 6c,d). Overall, cumulative datasets with these MIPs suggest this technique is a highly multiplexable capture system that can be utilized with multiple molecular detection assays for genetic variant identification.

## Discussion

Bacterial organisms frequently evolved innate immunity to antibiotics for survival in environmentally competitive niches^25^. Recently however ecological studies have shown development of antibiotic resistance in bacterial pathogens caused by increased antibiotic usage in animals, food, and humans^26^. A continuous cycle of newly acquired resistances results from the acquisition of genetic material or genetic variations in an organism’s genome. Currently, standard AR susceptibility testing is a laborious and lengthy process requiring pure bacterial cultures isolated from patient samples grown in the presence of antibiotics^27^. While susceptibility testing will likely remain the gold standard for AR confirmation, molecular techniques have arisen offering faster diagnosis to help guide therapeutic regimens^5^,^28^.

Real-time PCR is currently the standard molecular diagnostic for pathogen detection and more recently has been used for AR profiling^29^,^30^ although NGS is quickly being adopted in clinical laboratories. Real-time PCR is advantageous to other molecular diagnostics in many aspects including cost, time to answer, and sensitivity. One major aspect for time to answer is assay sensitivity and the ability to detect pathogen directly from patient samples without the necessity of culture. Bacteremia in septic patients can range more than 10-fold although<1000 colony forming units/ml of blood is common^31^,^32^. Real-time PCR offers sensitivities approaching one target copy per reaction^28^,^29^,^33^ allowing sample processing and detection directly from patient samples. NGS capabilities have outpaced Moore’s law boasting continuous technical improvements over the past decade however, relative to real-time PCR, diagnostic sensitivities are still lacking^9^. Metagenomic sequencing from clinical samples requires ultra-deep sequencing to obtain desired coverage levels of targeted pathogen and is not practical for routine clinical use due to cost and informatics burden. Targeted sequencing helps to bridge the gap between the sensitivities seen with real-time PCR and the quantitative data associated with NGS.

Analytical LODs for MIPs as an upfront amplification strategy for NGS fell within a range of approximately 1000 input genome copies. Mock clinical samples required an approximate 10-fold greater number of genome copies for positive detection above background levels. This difference is likely attributed to the carryover of inhibitors from complex matrices and variable efficiencies of sample processing methods which are known to affect PCR LODs^9^,^34^,^35^,^36^. Other factors affecting LOD include poor signal to noise and misidentification of pooled samples. Specifically, excess MIP backbone within each sample resulted in higher noise at low sample input concentrations (Supplemental Figure S1). *De novo* assembly of the un-mapped reads showed residual MIP backbone present after the cleanup. This was due to the formation of concatemers during the amplification process. The resulting background was more problematic as input concentrations approached the background cutoff. Similarly, incorrect assignment of dual indexed samples or sample bleeding^22^,^23^ was more pronounced at lower input concentrations. At these values the percentage of correctly mapped reads dropped below 50% for most capture regions tested. This read misidentification is an inherent limitation with multiplexed NGS which, as technology improves, can be overcome^23^.

While more sensitive, real-time PCR still suffers from an inability to significantly multiplex reactions. This is problematic with unknown samples which would require numerous screenings for identification and is not feasible with limited patient sample volumes. This is exacerbated in regards to AR which is multifaceted involving enzymes, pumps, and genetic variations requiring multiple target identifications^5^. Metagenomic sequencing could both classify the organism along with known resistance markers; however, the expertise and computing infrastructure required for analyzing data is still burdensome for clinical and biosurveillance programs. Metagenomic sequencing is not conducive for pooled sample NGS runs due to the difficulty obtaining adequate sequence coverage for genetic variants in complex matrices, increasing per sample costs. Depending on the NGS instrument, ultra-deep sequencing requires runs times ranging from days to over a week depending on desired coverage^9^,^37^. Targeted sequencing allows for significant coverage of desired regions over background genome and simple reference based mapping^9^. This permits sample pooling resulting in significant cost reduction with sequencing results in hours depending on desired amplicon length^38^. The cost burden of MIPs as an upfront amplification technology is marginal and increases NGS library preparation time by approximately 5–6 hours which is only slightly longer than multiplexed PCR based amplification technologies.

The requirement of a prior knowledge concerning the input etiologic agent or desired target region for probe design is an inherent caveat to targeted sequencing approaches; however, most new outbreaks such as Ebola virus or new resistant hospital acquired infections like carbapenem-resistant enterobacteriaceae have at least some representation within the public sequence repositories^39^,^40^. Even completely novel pathogens such as SARS coronavirus have some predicate within the phylogeny that allows for taxonomic placement based on targeted genomic sequence^41^. For bacterial identification specifically, 16S ribosomal RNA has been used to classify organisms for decades^42^. Conserved DNA stretches which flank hypervariable regions have been used for PCR amplification and could be adapted to MIPs for pathogen identification^43^,^44^. These probes along with viral specific targets would cover a significant percentage of human specific pathogens while new probes, due to the multiplex capabilities of MIPs, can be designed as completely novel organisms are classified by whole genome sequencing.

Efficient, specific, and reproducible multiplex capture and enrichment of DNA regions within an organism as well as across diverse species is an important part of a diagnostic or biosurveillance panel for targeted NGS. Techniques such as high level multiplex PCR often have reduced specificity when highly multiplexed due to non-specific amplification resulting from interactions in primer pairs^45^. Target circularization techniques such as MIPs have increased specificity attributed to 1) dual enzymatic events required for gap fill and ligation, 2) exonuclease digestion of non-circularized material, and 3) a common backbone which physically restricts the two homologous binding arms^45^. Sequencing data shown here demonstrated the ciprofloxacin MIP assay captured targeted regions of interest within the organism specifically and reproducibly at a high degree of multiplex. In fact, Pearson coefficients for intra-organism multiplex capture of targeted regions in *B. anthracis* and *F. tularensis* showed a high degree of correlation (Supplementary Table S2). Similarly, correlation analysis of inter-organism probes from all six capture regions was highly related and reproducible among experiments. This high degree of correlation suggests that the multiplexed probeset reproducibly captured regions of interest with similar efficiencies across a broad range of input DNA.

Diagnostic applicability of MIPs are not solely limited to NGS. MIPs also serve as a general enrichment strategy for other molecular detection technologies such as previously published HRM real-time PCR. Because these probes captured approximately 200 base pairs of genetic information, this allows quick identification for the presence or absence of known genetic variants that can later be characterized at higher granularity via NGS for other potential resistant conferring elements. This triaged approach becomes particularly vital when patient sample volumes are restricted, thereby limiting the application of multiple molecular diagnostic assays or non-multiplexed screening techniques. MIPs represent an overarching multiplexable amplification technology amenable with NGS technologies and other downstream molecular diagnostic techniques allowing for potential diagnostic applications including organism classification, gene identification, and genetic variant detection to name a few.

## Material and Methods

### Strains used and DNA preparation

Bacterial strains used in this study included wildtype and ciprofloxacin resistant Bacillus *anthracis* ΔANR, *Yersinia pestis* KIM5, and *Francisella tularensis* Schu4. *B. anthracis* wildtype and ciprofloxacin resistant mutant S3–8 clones were originally obtained from Dr. Lance B. Price^6^, *Y. pestis* wildtype and ciprofloxacin resistant mutant M1 clones were originally obtained from Dr. Luther Lindler^8^, and *F. tularensis* wildtype and ciprofloxacin resistance mutants were obtained from Dr. David Kulesh^4^. DNAs were extracted and purified using the Qiagen EZ1 kit (Qiagen, Valencia, Ca) according to the manufacturer’s instructions. For Figs 3 and 6,WGA DNA was used and WGA was performed utilizing Qiagen’s REPLI-g-Midi Kit according to manufacturer’s protocol. For Fig. 6, 1 μg of WGA DNA was spiked into 1 ml of whole blood (BioreclamationIVT, Baltimore, MD) and 10 fold serially diluted. 100 μl of each dilution was extracted using TRIzol LS (Thermo Fisher Scientific, Waltham, MA) and the EZ1 (Qiagen) according to the manufacturer’s instructions For non-template controls water or, in Fig. 6, non-spiked extracted whole blood was used in lieu of bacterial DNA. DNA concentration was quantified utilizing Qubit dsDNA BR and HS assay kits (Thermo Fisher Scientific).

### MIP identification and design

MIP probes were designed by Bioinnovation Solutions (Lausanne, Switzerland) to flank SNPs and deletions known to confer ciprofloxacin resistance and purchased by USAMRIID. Briefly, *B. anthracis* AMES *gyrA, gyrB,* and *parC* (GenBank NC_003997.3), *Y. pestis* A1122 (GenBank NC_017168.1), and *F. tularensis gyrA* and *parE* (GenBank AJ749949.2) genes were used as BLAST templates. Consensus sequence alignments with the highest *E*-value hits were then used to identify conserved regions for probe hybridization. Based on these conserved regions, 24 probes targeting *B. anthracis*, 12 targeting *Y. pestis*, and 8 targeting *F. tularensis* were designed. All probes were designed to capture sequences ranging from 180–210 bp. Probes were synthesized by Integrated DNA Technologies (IDT, Coralville, IA). Complimentary probe arms are represented in Supplementary Table S1.

### Bacterial MIP probe analysis

Probes were re-suspended in buffer TE and pooled to a final per probe concentration of 3 nM. A total of 44 probes were combined and used as a master probe mix. Probe capture protocol was performed as previously described with slight modifications^20^. The modified protocol is as follows: Total probe mix was hybridized to 10-fold serial dilutions of WGA or non-amplified extracted DNA samples (see above) of *B. anthracis, Y. pestis*, and *F. tularensis* by adding 3 nM probe to 1.5× ampligase buffer (Epicentre, Madison, WI), heat to 94 °C for 2 minutes, and then ramped to 60 °C (0.1 °C/sec temperature decrease) with a 60 min hold at 60 °C. Gap filling and ligation were performed by the addition of Phusion high-fidelity PCR master mix with HF buffer (New England Biolabs, Ipswich, MA), 4 units Ampligase (Epicentre), ampligase buffer, and 0.4 μM dNTPs (BioFire, Salt Lake City, UT). The reaction was then incubated at 60 °C for 60 min and held at 15 °C.

Non-circular DNA was removed by exonuclease I and exonuclease III digestion. Specifically, the reaction was heated to 94 °C for 2 min and held at 37 °C while 10 units of exonuclease I and 50 units of exonuclease III were added. Samples were then incubated at 37 °C for 30 min followed by enzymatic inactivation at 94 °C for 15 min. Capture sequence was then amplified by the addition of Platinum *taq* (Thermo Fisher Scientific), 0.4μm forward 5′-CAGATGTTATGCTCGCAGGTC and reverse 5′-GGAACGATGAGCCTCCAAC primers, 1.0× buffer with 50mm MgCl_2_, and 0.4 μM dNTPs. Reactions were amplified at 95 °C for 3 min, 40 cycles of 95 °C for 30 sec, 60 °C for 30 sec, 72 °C for 1 min, and a hold of 72 °C for 10 min. Following amplification samples were purified utilizing Agencourt AMPure XP beads (Beckman Coulter, Pasadena, Ca) per the manufactures protocol with the slight alteration to a 1:1 mixture of bead to sample volume.

### Sequencing and analysis

Library preparation was performed on the Apollo 324 system (Wafergen Biosystems, Fremont, CA) utilizing the PrepX ILM 32i DNA library kit (Wafergen Biosystems) and TruSeq dual indexes (Illumina, San Diego, CA) according to the manufacturer’s instructions. Samples were then enriched with the TruSeq HT preparation kit (Illumina) and purified as described above. Samples were quantified with the Qubit dsDNA BR and HS assay kit (Thermo Fisher Scientific) and pooled based on total concentration. Proper adaptor ligation confirmation and final normalization were performed using the KAPA library quantification kit (Kapa Biosystems, Wilmington, MA).

Amplicons were sequenced using the MiSeq platform (Illumina) using the 2 × 150 cycle sequencing kit. Sequencing reads were analyzed using CLC Genomic Workbench (CLC Bio, Cambridge, MA). Reads were trimmed for quality, and the universal primer sequences were used to filter proper capture sequences. The trimmed and filtered reads were then mapped against known reference genes including *B. anthracis* AMES *gyrA, gyrB,* and *parC* (GenBank NC_003997.3), *Y. pestis gyrA* (GenBank NC_003143.1), and *F. tularensis gyrA* and *parE* (GenBank AJ749949.2). Standard settings for read mapping were used and required 70% of the read length matching the reference with at least 80% identity while ignoring non-specific read mappings for positive hits. Non-template controls were run concurrently to measure sample bleeding which occurs when sequencing reads are misidentified during the demultiplexing process. A cutoff was determined as the average number of sequencing reads mapped to the reference gene in the non-template controls plus three times the standard deviation. 30X coverage represents the point at which there is 30 times the read coverage above this cutoff control. LODs were determined based on a defined 30× read coverage at an indicated genetic variant. Variant analysis was performed on mapped sequencing reads using a minimum read coverage of 5× and minimum variant frequency of 10%. Linear regression and Pearson correlations were performed using GraphPad Prism v. 6.04 graphing software (GraphPad, La Jolla, CA).

### High Resolution Melt Assays

Amplicons resulting from the MIP capture protocol in Fig. 4 before NGS library preparation were used as templates and diluted 1:10,000 for high-resolution melt analysis. This was compared to concentrations of genomic DNA which were used as an input to the MIP protocol. HPLC purified primers for *F. tularensis gyrA* (forward 5′-CGGTAAATATCACCCTCATGGAG and reverse 5′- AGGTTGTGCCATTCTGACAATAGTAT) and *parE* (forward 5′-CTTACATGGCATTTTGAAACTGGAC and reverse 5′- CAGCTTCTAGTTTATGGTCAAGATAGCC) were utilized^4^. Reaction conditions included 1X Lightcycler 480 High Resolution Melting Master Mix (Roche Diagnostics, Indianapolis, IN), 0.4 μM forward and reverse primers, and MgCl_2_ to a final concentration of 3 mM. Reactions were performed on a Roche 480 Lightcycler (Roche Diagnostics, Indianapolis, IN) with thermocycling conditions consisting of a pre-incubation cycle of 95 °C for 10 min, a 45 cycle amplification step of 95 °C for 10 sec, 60 °C for 10 sec, and 72 °C for 10 sec with a fluorescence reading taken at the end of each 72 °C step, and a high-resolution melt step of 95 °C for 1 min, 45 °C for 1 min, and melt curve from 65 °C to 95 °C with 25 fluorescent readings taken per 1 °C temperature change. Values were generated using the melt curve genotyping analysis with the integrated Roche LightCycler 480 software version 1.5.1.

## Additional Information

**How to cite this article**: Stefan, C. P. *et al*. Targeted next-generation sequencing for the detection of ciprofloxacin resistance markers using molecular inversion probes. *Sci. Rep.* **6**, 25904; doi: 10.1038/srep25904 (2016).

## Supplementary Material

Supplementary Information

## Figures and Tables

**Figure 1:**
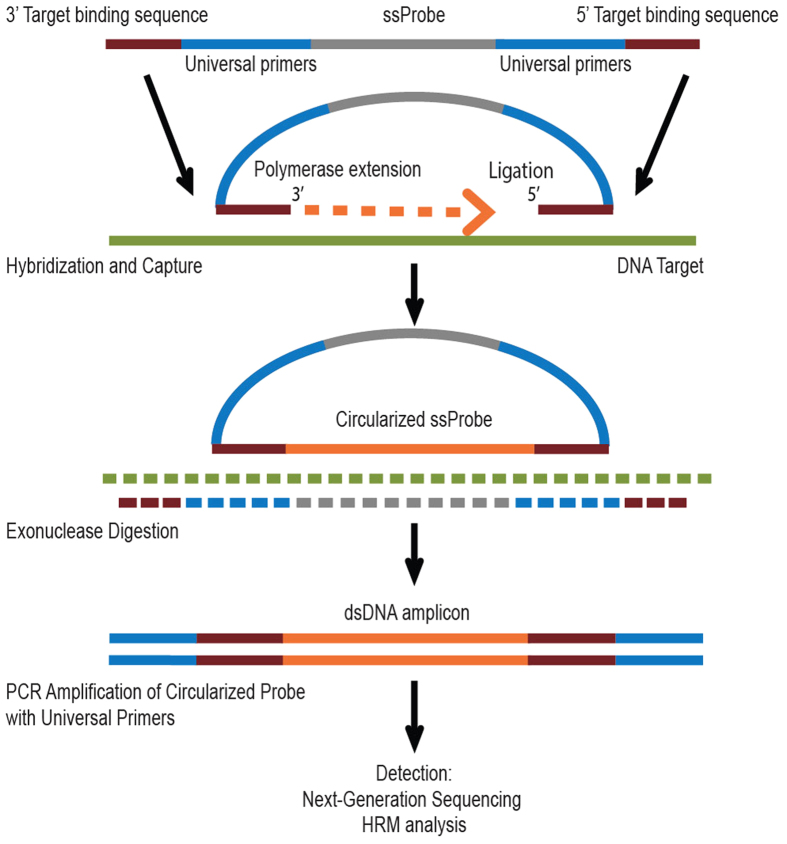
Molecular inversion probe flow-chart. MIPs are single stranded oligonucleotides with a common backbone and universal primers bordered by capture arms designed to complement regions of interest. After probe hybridization the subsequent sequence gap is filled with a polymerase step and closed by a ligase. Exogenous host DNA and linear probes are digested with exonucleases leaving single stranded circularized probes. The capture region is then amplified utilizing the universal primers, creating approximately 200 base pair double stranded amplicons ready for detection with multiple molecular diagnostic platforms.
