# Supplementary material for: Targeted next-generation sequencing for the detection of ciprofloxacin resistance markers using molecular inversion probes
Source: Sci Rep. 2016 May 13;6:25904. doi: 10.1038/srep25904 (PMC4865750; doi:10.1038/srep25904)

Targeted next-generation sequencing for the detection of ciprofloxacin resistance markers using molecular inversion probes

Christopher P. Stefan[1], Jeffrey W. Koehler[1], Timothy D. Minogue[1*]

[1] United States Army Medical Research Institute of Infectious Disease, Diagnostic Systems Division, Fort Detrick, Maryland, 21702, United States of America

christopher.p.stefan.ctr@mail.mil

jeff.w.koehler.ctr@mail.mil

[*]timothy.d.minogue.civ@mail.mil

Table S1. Sequences of capture probe arms

Table S2. Pearson's correlation demonstrates significant relatedness between capture regions

Pearson's correlation matrix coefficients for the linear range of every pair of target regions from Figure 3, Figure 4, and Figure 6 are represented. Blue indicates data from Figure 2, orange indicates data from Figure 5, and green indicates data from Figure 6.

Table S3. Linear regression analysis results for Figure 4.

A linear regression analysis was performed on the linear range of genetic variant coverage versus number of input genomes for Figure 4. R squared, slope, and equation along with the p-value for relatedness is given.

Table S4. Total number of sequencing reads after quality trim

Table S4 provides the total number of sequencing reads obtained after the quality control trim for Figures 3, 4, 5 and 6.

Figure S1. Percentage of reads from probe challenge across a broad range of input DNA concentrations

MIP probsets were pooled and tested against 10-fold dilutions of WGA amplified input DNA from wild-type (a) *B. anthracis*, (b) *Y. pestis,* and (c) *F. tularensis.* All samples were dual indexed and pooled before sequencing on the Illumina MiSeq platform. The average percentage of mapped reads for each reference gene along with the unmapped read percentage versus the estimated total input genomes is plotted. Other represents non-mapped reads including MIP backbone and any mammalian contamination.

Supplementary Table S1

	Organism	Gene	5' complimentary arm	3' complimentary arm
1	*B. anthracis*	*gyrA*	TGAGGGTGATACTTACCGATTACTT	TTCAGAACCATCATAGTTATCTTG
2			CCATGAGGGTGATACTTACCGATT	TCTTTCAGAACCATCATAGTTATCT
3			CACCATGAGGGTGATACTTACCG	ACACAATCGGCTCTCTTTCAG
4			TCACCATGAGGGTGATACTTACCG	TCAGAACCATCATAGTTATCTTGA
5			ATCACCATGAGGGTGATACTTACCG	ATCGGCTCTCTTTCAGAACC
6			AACAGCTGAATCACCATGAGGGT	CTCTCTTTCAGAACCATCATAGT
7		*gyrB*	ACGCGCTCTTACGACGTGTC	GCAGTAATAATTGTACGCACTTC
8			ACTTGAAACTTCTAACGCGCTCT	TGCAGTAATAATTGTACGCACT
9			AATTTACCAGGTAAACTTGAAACTTCT	AATTGCAGTAATAATTGTACGCA
10			ATCTGCTAATTTACCAGGTAAACTTGA	ATAACTTTATGATAACGAGCTTTCT
11			AAGAGCAATCTGCTAATTTACCAGG	CGTCATAATAATAACTTTATGATAACGA
12			TGAAGAGCAATCTGCTAATTTACCAG	GTCATAATAATAACTTTATGATAACGAG
13			CACCCTCTACAATGTAAATTTCACTAA	GTACGAATATGCGCACCATCT
14			CCGGCAGAGTCACCCTCTACA	GATAGAAGAACGTTAATAATAGGGTA
15		*parC*	TCATGTACGTTTCCTTCTACATACAT	CAATTGGTGATAATCGGGCTTC
16			ATCATGTACGTTTCCTTCTACATACA	TTGGTGATAATCGGGCTTCC
17			ACCAATTGCATCTGAGTTATTACGT	AAACGTACATGATAAAGCGTTC
18			GATGGTACGTTTAAGTCAAACTTGG	TGAAGACGTGTTAGGTGACC
19			AAGCGATGGTACGTTTAAGTCAAAC	ACATGCAAGCAGAGAAGTTTC
20			TATATGAAGCGATGGTACGTTTAAGT	GTGGAATAACATGCAAGCAGA
21			CGTTATTGGTAACTATCACCCGCA	AGAGAAGTTTCATGACCTCCC
22			ACGTTATTGGTAACTATCACCCGC	GGAGGTGGAATAACATGCAAG
23			TAACGTTATTGGTAACTATCACCCG	GCAAGCAGAGAAGTTTCATGA
24			GTAACGTTATTGGTAACTATCACCCG	CGGAGGTGGAATAACATGCA
25	*Y. pestis*	*gyrA*	TGTCACCATGCGGGTGGTATTT	ATTCTGGTCGGCATAACAGC
26			CTGTCACCATGCGGGTGGTATT	GTTTACCAGCAGGTTAGGGATT
27			CGCGCTGTCACCATGCGGG	CGTTTACCAGCAGGTTAGGG
28			GCAGGGTAACTTCGGTTCCGT	ACGTGCGTTACCAGATGTC
29			GGGCAGGGTAACTTCGGTTCC	CGTGATGGACTGAAACCGG
30			TGGGCAGGGTAACTTCGGTTCC	GATTATGCGATGTCCGTTATTG
31			ATGGGCAGGGTAACTTCGGTTC	AGATGTCCGTGATGGACTGA
32			GGATGGGCAGGGTAACTTCGG	CGTGCGTTACCAGATGTCC
33			TGGATGGGCAGGGTAACTTCGG	GACGTGCGTTACCAGATGT
34			TGGTGGATGGGCAGGGTAACTT	TGGATTATGCGATGTCCGTTA
35			GCCGTTCTCACTGCGCTATATG	TGCGATGTCCGTTATTGTCG
36			AGCGCGGTCTACGACACTATC	CAGAGAAATAACACCGGTCAAC
37	*F. tularensis*	*gyrA*	TGATTCTCCTGCAGCAAT	GCAATGAATGAACTATCAAA
38			GCTATACGCTAGTAGATGGA	TGTGCGTGATGGTCTTAAGC
39			ACGCATGAGCTCTTGATCGA	TGCAAGGGTTGTCGGTGA
40			CACAACCTTTCTCATTGCGCT	TGGTCGTGCTTTGCCAGA
41	*F. tularensis*	*parE*	GTGAAGATCTTAGTGAAAGT	GATACTTTGTAAGGCTC
42			GTATCAAGAATAGCTATG	CACAATAAAATATTCAA
43			GATCTTAGTGAAAGTATCA	GAGAAGCTTACATGGCAT
44			TGCAGTATTTTGCTGGTGTGA	GCTTGAAGCAAAAGCGATACT

Supplementary Table S2

		Figure 3						Figure 4							Figure 6					
		B.a. gyrA	*B.a. gyrB*	*B.a. parC*	*Y.p. gyrA*	*F.t. gyrA*	*F.t. parE*	*B.a. gyrA* C254T	*B.a. gyrB* G1294A	*B.a. parC* C242T	*Y.p. gyrA* C254T	*F.t. gyrA* C248T	*F.t. gyrA* G259T	*F.t. parE* □TTAAA	*B.a. gyrA*	*B.a. gyrB*	*B.a. parC*	*Y.p. gyrA*	*F.t. gyrA*	*F.t. parE*
Figure 3	*B.a. gyrA*		1.000	0.976	0.999	0.992	0.993	0.999	0.989	0.996	0.993	0.995	0.995	1.000	0.998	0.992	0.996	0.980	0.999	0.997
	B.a. gyrB	1.000		0.979	0.998	0.994	0.995	0.999	0.987	0.994	0.991	0.994	0.994	1.000	0.997	0.990	0.997	0.977	0.998	0.998
	B.a. parC	0.976	0.979		0.966	0.995	0.994	0.968	0.933	0.951	0.943	0.950	0.950	0.974	0.959	0.939	0.991	0.913	0.965	0.990
	Y.p. gyrA	0.999	0.998	0.966		0.986	0.987	1.000	0.995	0.999	0.997	0.999	0.999	0.999	1.000	0.996	0.991	0.988	1.000	0.992
	F.t. gyrA	0.992	0.994	0.995	0.986		1.000	0.987	0.963	0.976	0.970	0.975	0.975	0.991	0.982	0.968	0.999	0.948	0.986	0.999
	F.t. parE	0.993	0.995	0.994	0.987	1.000		0.988	0.965	0.978	0.972	0.977	0.977	0.992	0.983	0.970	0.999	0.950	0.987	0.999
Figure 4	*B.a. gyrA* C254T	0.999	0.999	0.968	1.000	0.987	0.988		0.994	0.998	0.996	0.998	0.998	1.000	1.000	0.995	0.992	0.986	1.000	0.993
	B.a. gyrB G1294A	0.989	0.987	0.933	0.995	0.963	0.965	0.994		0.998	1.000	0.999	0.999	0.990	0.997	1.000	0.972	0.999	0.995	0.974
	B.a. parC C242T	0.996	0.994	0.951	0.999	0.976	0.978	0.998	0.998		1.000	1.000	1.000	0.997	1.000	0.999	0.984	0.994	0.999	0.985
	Y.p. gyrA C254T	0.993	0.991	0.943	0.997	0.970	0.972	0.996	1.000	1.000		1.000	1.000	0.994	0.998	1.000	0.978	0.997	0.997	0.980
	F.t. gyrA C248T	0.995	0.994	0.950	0.999	0.975	0.977	0.998	0.999	1.000	1.000		1.000	0.996	1.000	0.999	0.983	0.995	0.999	0.984
	F.t. gyrA G259T	0.995	0.994	0.950	0.999	0.975	0.977	0.998	0.999	1.000	1.000	1.000		0.996	1.000	0.999	0.983	0.995	0.999	0.984
	F.t. parE □TAAA	1.000	1.000	0.974	0.999	0.991	0.992	1.000	0.990	0.997	0.994	0.996	0.996		0.998	0.993	0.995	0.982	0.999	0.996
Figure 6	*B.a. gyrA*	0.998	0.997	0.959	1.000	0.982	0.983	1.000	0.997	1.000	0.998	1.000	1.000	0.998		0.998	0.988	0.991	1.000	0.989
	B.a. gyrB	0.992	0.990	0.939	0.996	0.968	0.970	0.995	1.000	0.999	1.000	0.999	0.999	0.993	0.998		0.976	0.998	0.996	0.978
	B.a. parC	0.996	0.997	0.991	0.991	0.999	0.999	0.992	0.972	0.984	0.978	0.983	0.983	0.995	0.988	0.976		0.959	0.991	1.000
	Y.p. gyrA	0.980	0.977	0.913	0.988	0.948	0.950	0.986	0.999	0.994	0.997	0.995	0.995	0.982	0.991	0.998	0.959		0.988	0.961
	F.t. gyrA	0.999	0.998	0.965	1.000	0.986	0.987	1.000	0.995	0.999	0.997	0.999	0.999	0.999	1.000	0.996	0.991	0.988		0.992
	F.t. parE	0.997	0.998	0.990	0.992	0.999	0.999	0.993	0.974	0.985	0.980	0.984	0.984	0.996	0.989	0.978	1.000	0.961	0.992	

Supplementary Table S3

	Slope	R square	Equation
B.a gyrA C254T	1.374 ± 0.1152	0.9343	Y = 1.374*X - 2.519
B.a gyrB G1294A	1.162 ± 0.06236	0.972	Y = 1.162*X - 2.087
B.a parC C242T	1.190 ± 0.1031	0.9301	Y = 1.190*X - 1.766
Y.p gyrA C254T	1.203 ± 0.09160	0.9452	Y = 1.203*X - 1.991
F.t gyrA C248T	1.120 ± 0.06865	0.9638	Y = 1.120*X - 1.402
F.t gyrA G259T	1.144 ± 0.08039	0.953	Y = 1.144*X - 1.516
F.t parE TTAAA	1.076 ± 0.08997	0.9347	Y = 1.076*X - 0.9561

Supplementary Table S4

Figure # 3		Total number of sequencing reads after quality trim									
		B. anthracis			*Y. pestis*			*F. tularensis*			
	Replicates	A	B	C	A	B	C	A	B	C	**NTC**
Total Input Genomic DNA (ng)	0.00005	169,215	178,286	151,733	198,354	136,300	246,053	130,378	148,719	137,034	137034
	0.0005	178,900	227,456	213,158	202,080	189,578	197,523	150,044	152,636	119,617	119617
	0.005	179,643	163,978	210,457	173,344	190,771	223,511	174,249	176,419	112,557	112557
	0.05	128,902	132,211	145,998	125,945	114,748	156,475	133,364	163,605	132,137	132137
	0.5	145,322	171,972	206,249	177,774	159,143	176,306	146,468	187,414	121,281	121281
	5	189,185	207,220	208,264	195,941	138,260	160,041	267,755	225,965	157,718	157718
	50	260,864	253,007	218,900	324,539	161,145	224,737	240,901	257,879	253,128	253128
	500	233,881	220,628	233,228	271,252	209,564	223,742	301,039	289,538	349,516	349516

Figure # 4		Total number of sequencing reads after quality trim									
		B. anthracis			*Y. pestis*			*F. tularensis*			
	Replicates	A	B	C	A	B	C	A	B	C	**NTC**
Total Input Genomic DNA (ng)	0.0005	311,481	350,364	339,137	354617	184453	338141	311,948	337,432	181,757	240,323
	0.005	198,337	375,453	347,685	199167	166640	322022	207059.00	186372.00	244,599	219,305
	0.05	195,412	310,271	300,265	286931	291553	205106	282028.00	195679.00	207,721	181,494
	0.5	226,723	221,430	204,997	143826	240562	253603	264,650	346,270	431,639	

Figure # 5	Total number of sequencing reads after quality trim							
	Replicates							
	1	2	3	4	5	6	7	8
B. anthracis	196,847	218,465	201,816	191,804	200,830	211,984	196,994	194,396
Y. pestis	207,200	261,827	234,880	196,676	209,070	231,335	202,331	178,947
F. tularensis	228,611	306,955	298,762	238,461	277,936	227,738	271,381	246,695
	Replicates							
	9	10	11	12	13	14	15	16
B. anthracis	218,940	274,152	198,832	179,452	226,599	218,409	225,565	205,022
Y. pestis	281,838	281,800	254,382	256,554	214,718	278,058	281,390	259,211
F. tularensis	284,853	308,182	269,649	241,014	283,564	255,101	261,691	236,419

		Total number of sequencing reads after quality trim									
		B. anthracis			*Y. pestis*			*F. tularensis*			
	Figure # 6	A	B	C	A	B	C	A	B	C	**NTC**
Spike (ng/ml of blood)	1000	127,922	127,351	158,844	172,648	208,105	176,023	186,039	205,931	296,388	189,043
	100	118,676	110,206	158,740	150,889	195,642	177,254	224,088	176,736	213,073	198,478
	10	101,428	110,801	119,379	135,581	130,011	192,618	144,801	190,951	186,376	168,716
	1	101,564	149,206	112,612	157,007	204,065	205,928	143,869	240,484	144,178	
	0.1	98,906	124,798	178,476	173,780	124,542	182,914	163,837	209,215	183,106	
	0.01	96,218	124,778	166,603	138,243	199,777	165,571	171,691	166,583	2,111,645	

Supplementary Figure S1

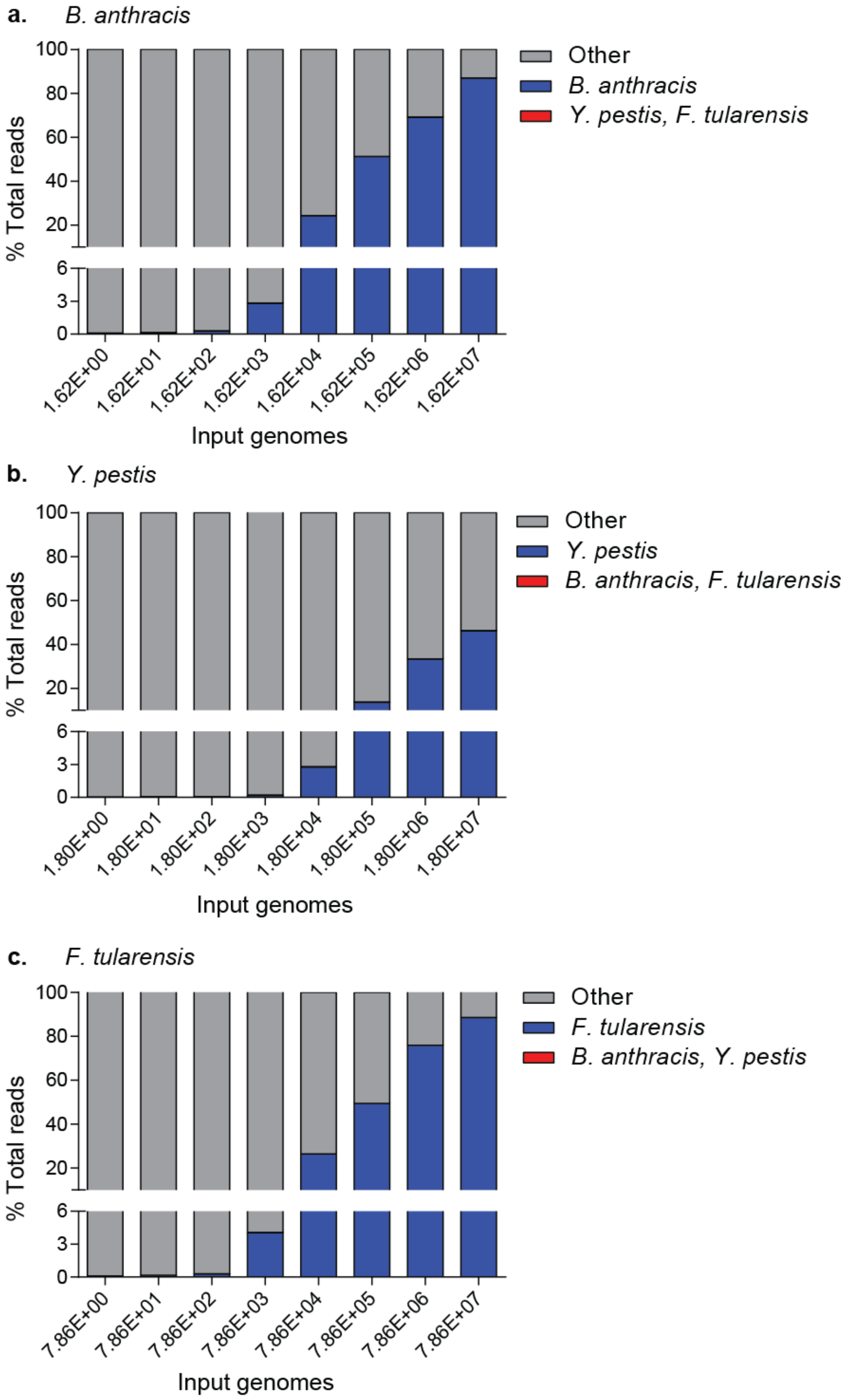

Supplement: Supplementary Information [file srep25904-s1.pdf]
